# The addiction behavior of short-form video app TikTok: The information quality and system quality perspective

**DOI:** 10.3389/fpsyg.2022.932805

**Published:** 2022-09-06

**Authors:** Yao Qin, Bahiyah Omar, Alessandro Musetti

**Affiliations:** ^1^School of Communication, Universiti Sains Malaysia (USM), George Town, Penang, Malaysia; ^2^Department of Humanities, Social Sciences and Cultural Industries, University of Parma, Parma, Italy

**Keywords:** TikTok, information quality, system quality, flow, addiction, S–O–R model, quantitative research

## Abstract

TikTok has one of the most advanced algorithm systems and is the most addictive as compared to other social media platforms. While research on social media addiction is abundant, we know much less about how the TikTok information system environment affects users’ internal states of enjoyment, concentration, and time distortion (which scholars define as the flow experience), which in turn influences their addiction behavior. To fill this gap, this study collects responses from 659 adolescents in China aged between 10 and 19 years old, and the data is then analyzed using Partial Least Square (PLS). We find that the system quality has a stronger influence than information quality in determining adolescents’ experience with TikTok and that the flow experience has significant direct and indirect effects on TikTok addiction behavior. Notably, this study finds that TikTok addiction is determined by users’ mental concentration on the medium and its content. Several theoretical insights from the stimulus–organism–response (S–O–R) model and the flow theory are used to explain the findings.

## Introduction

With the progress in information technology and the change in preferences of network users, new social media are popping up every day. Originated in 2014, short videos have received favor in recent years. Due to its “short” nature (usually less than 60 s), the short video is very suitable for playing on mobile terminals and convenient for users to share on various social platforms. Led by TikTok (Douyin), the short video quickly captures the hearts of all age groups (Du et al., 2020) and has also become the most popular social media platform among millennials in China (Jung and Zhou, 2019).

TikTok enables users to capture memorable moments and create short-form videos to record their lives. It brings lots of entertainment to people but is also becoming a new form of social media addiction (Smith and Short, 2022). The term, social media addiction, refers to the consistency of addiction-like symptoms or a lack of self-discipline regarding social media (Casale et al., 2018; Klobas et al., 2018; Tarafdar et al., 2020). Literature on social media addiction has focused disproportionately on Facebook, Instagram, and other mature social media platforms while ignoring concerns about TikTok’s penetration and related maladaptive behavior (Smith and Short, 2022).

Examining TikTok addiction behavior is important because of several reasons. First, TikTok is one of the fastest-growing apps and has surpassed previous social media in terms of user numbers and usage intensity (Montag et al., 2021). Second, TikTok has the most advanced algorithm system, especially in terms of participation, content, and types of interaction, which makes the addiction problem of TikTok more severe than the other popular social media (Zhang X. et al., 2019; Iram and Aggarwal, 2020; Zhao, 2021; Smith and Short, 2022). While the underlying adverse symptoms of addiction are similar across different platforms, the intensity and driving factors of TikTok addiction are unique (Smith and Short, 2022). Third, TikTok’s target audiences are adolescents and young adults with short attention spans. Based on the data statistics of TikTok penetration in China, the largest group of users is 6–17 years old, accounting for 31.59%, followed by 18–24 years old (30.14%), 25–30 years old (20.85%), 31–35 years old (8.66%), and over 35 years old (8.76%) (Mou, 2020). This has raised serious concern as TikTok addiction affected young people seriously. They are naive and easily absorbed when exposed to a wide variety of short video contents (Weimann and Masri, 2020).

Given the complexity of addiction behavior, scholars generally acknowledge a closed-loop relationship between TikTok addiction and algorithm optimization (Zhao, 2021). Users seem to be caught in an entertainment spiral. Studies on social media addiction have examined some external factors causing addiction behavior such as technical factors (Gao L. et al., 2017; Hasan et al., 2018). There are also some evidences from past studies suggesting the influence of flow experience—an inner feeling of enjoyment, concentration, and time distortion—in determining users’ addiction behavior (Zhao and Zhou, 2021). The indirect influence of flow experience is also found in the context of online gaming addiction (Park and Hwang, 2009). Despite the growing body of literature on social media addiction (D’souza, 2019; Leong et al., 2019; Cao et al., 2020), several kinds of research concentrated on either internal factors (psychological positive reinforcement, such as enjoyment) (Sun et al., 2015; Gao W. et al., 2017; Lin et al., 2020) or external factors (such as internet and technology) (Tian et al., 2017; Cao et al., 2020) that lead to addiction behavior. However, the antecedent of users’ internal perception was mostly ignored.

To gain a general and more comprehensive understanding of addiction behavior, this study examines the effects of TikTok technical environment factors (i.e., information quality and system quality) on adolescents’ inner perception of online experience (i.e., flow experience, which consists of enjoyment, concentration, and time distortion), which in turn caused TikTok addiction behavior. This study adopts the stimulus–organism–response (S–O–R) model based on environmental psychology (Mehrabian and Russell, 1974) and flow theory (Csikszentmihalyi, 1975) to systematically expounds on adolescent addiction behavior in China. The study contributes to the communication literature by extending the S–O–R model and providing new insight by using TikTok’s technical environment to explain users’ online experience.

## Theoretical background and hypothesis development

### Stimulus–organism–response framework

The S–O–R model, also known as the environmental psychological model, was developed by Mehrabian and Russell (1974). The S–O–R framework proposed that the environmental aspects, which were regarded as environment stimulus (S), work together to affect people’s internal state (O), for example, psychological, cognitive, emotional, and intellectual internal states, which finally leads to specific execution of individual response (R) (Mehrabian and Russell, 1974; Moon et al., 2016). According to the S–O–R paradigm, the external environmental stimulus extended from the physical environment (such as technology and layout) to the individual inner state (cognitive and affective), causing an approach or avoidance response (Tuerlan et al., 2021).

The S–O–R model can be used to predict the impact of a specific environment on the emotional state and behavior of humans using social media (Luqman et al., 2017; Min et al., 2020), such as forecasting continuous or discontinuous intentions by identifying the influence of information system related environment on account of positive or negative perceptions (Cao and Sun, 2018; Zhao et al., 2020). Given the critical role of the information system technological environment in influencing users’ online behavior, we proposed that environmental stimulus (e.g., information quality and system quality) could influence Chinese adolescents’ inner state and provoke addiction behavior to TikTok.

### Information and system quality as stimulus (S)

The stimulus factor referred to the outer environment that the users faced (Zhu et al., 2020). It can be conceptualized as something that triggered or aroused action or increased action (Mehrabian and Russell, 1974). As an external factor, the stimulus was considered to have a great influence on the internal state of consumers, including various aspects, such as environmental, symbols, format, content, technical and spatial characteristics of information systems, etc. (Bagozzi, 1986; Suh and Prophet, 2018).

The concepts of information quality and system quality could be traced back to DeLone and McLean’s Information Systems Success Model (DeLone and McLean, 2003). In this model, information and system quality were two vital constructs. It was crucial in the context of social media since users’ perception of quality directly drove their subsequent behavior (Cheng, 2019). Previous research defined users’ perception of quality as their assessment of functions which reflected the overall performance of the application (Suhartanto et al., 2017; Li and Shang, 2020). Scholars regarded information quality as the representative of products or services, while system quality referred to the technological aspect. These two can be treated as environmental stimuli (Yen et al., 2018), and were used to explain the relationship between technological attributes, users’ internal state, as well as their subsequent behavior (Cao et al., 2020). Therefore, the quality of the information system was a critical issue when evaluating how users’ normal use of a platform gradually became addiction behavior.

The researchers detected some dimensions to measure information quality and system quality. For instance, Zhang et al. (2016) conducted research on Microblog, and considered information quality as a multidimensional concept, including reliability, timeliness, conciseness, and subscription, while system quality was regarded as a multidimensional concept, containing autonomy, broadcasting, interoperability, and ease of use. Nelson et al. (2005) used completeness, accuracy, format, and currency to measure the information quality, while used accessibility, reliability, response time, flexibility, and integration to measure system quality.

In the context of the short-form video application TikTok, this study adopted a suitable measurement instrument for TikTok’s information and system quality based on previous research. More specifically, conciseness, subscription, and usefulness were used to measure information quality, while flexibility, integration, ease of use, and response time were used to measure system quality. TikTok was equipped with advanced algorithm techniques for video recommendations of users. All the videos that users browsed depended on the system recommendation. TikTok system accurately calculated what each user was interested in based on a series of data mining, such as the user’s login method, the network-associated personal information, and the content of likes. These information contents were of high quality and could meet users’ appetites. Therefore, high information and system quality can provide a great online experience for users (Al-Fraihat et al., 2020).

### Flow experience as users’ internal states (O)

The term “organism” represented the internal state of individuals’ perception, feelings, and thinking (Bagozzi, 1986), which was composed of cognition and affection and mediated the relationship between the stimulus and individual responses (Mehrabian and Russell, 1974). Researchers considered the organism as a combination of emotion and cognition (Donovan and Rossiter, 1982). The cognitive state was the psychological process of individual information acquisition, processing, retention, and retrieval. Emotional states reflected the full range of emotions, including various feelings such as enjoyment, concentration, self-absorption, boredom, anxiety, disgust, etc. (Holbrook and Hirschman, 1982).

Based on this, Mehrabian and Russell (1974) proposed a hedonic–arousal–dominant (P–A–D) emotional state model, in which people’s subsequent controlling behavior or out-of-control behavior depends on the stimulus from the environment (Russell and Pratt, 1980). Although this model had been applied in many studies, the contexts and the consumers were different, and their inner perceptions might vary; thus, there was still disagreement about the interpretation of these three dimensions (Sun et al., 2021).

In the context of the TikTok user experience, the online information technology-based communication enhanced the psychological state of users’ enjoyment, concentration, and time distortion feelings (Lee et al., 2018), which were the critical components of the online flow experience (Lee et al., 2017). The flow was proposed by Csikszentmihalyi (1975) and regarded as intrinsic motivation, the inner sense of participating in an activity for pleasure feelings (Teo et al., 1999). In the subsequent study of the virtual network environment, the flow was defined as the user’s obsession, immersion, and deep participation in the use of technology (Kim et al., 2017), which offered useful insights for analyzing online users’ online experiences (Hoffman and Novak, 2009; Ding et al., 2010). Therefore, the flow played an important role in evaluating online information quality, system quality, and satisfaction (Gao L. et al., 2017), and was considered a valuable tool to identify users’ experience of the platform (Kim et al., 2020). Previous research also confirmed the role of excessive flow in determining users’ addiction behavior (Sun et al., 2015; Lin et al., 2020).

### TikTok addiction behavior as response (R)

The final behavior was the response in the S–O–R framework, including access and avoidance attitude or behavior (Mehrabian and Russell, 1974). The attitude could be generated from external environment stimulus or could be formed by organisms. Accessing behavior was a positive response and avoidance behavior was the counterpart. Chopdar and Balakrishnan (2020) pointed out in their research on the user behavior of mobile commerce applications that when users appreciated the value of the application and were satisfied with its user experience, purchase intention can be induced. Luqman et al. (2017) applied this model to discontinue the intention of SNS and believed that the excessive use of the online environment (i.e., excessive hedonic use) could cause sensory stimulation in users, thus leading users to give up on SNS. As a result, consumers with a good experience were more likely to continue using online applications.

In the context of the short-form video TikTok application environment, users were exposed to many technical characteristics or advanced functions, which can provide them with high information and system quality, such as interesting functions and personalized videos, which were full of addictive entertainment value. Thus, the integration of environmental stimulus and internal organism triggered the specific TikTok addiction behavior. This study regarded addiction behavior as part of the adverse consequences of flow experiences associated with the use of TikTok.

Young (1999) proposed a set of comprehensive instruments to measure addiction behavior, which was adapted and translated into a Chinese version to apply in China (Yu and Fu-min, 2005; Zhang X. et al., 2019). The Chinese version of measurement tools exhibited good performance in Chinese adolescents (Liang et al., 2016; Zhang et al., 2018, Zhang R. P. et al., 2019). This study also adopted this measurement, specifically, adopting the Chinese version of Yu and Fu-min (2005) to measure TikTok addiction behavior.

## Research model and hypotheses

**FIGURE 1:**
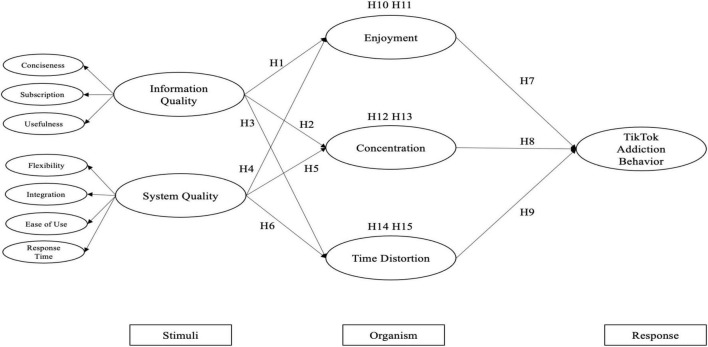
The research model.

Drawing upon the theory of the S–O–R paradigm and Flow theory, we proposed a theoretical-based model to explore the influencing factors for TikTok addiction behavior. The S–O–R paradigm laid out primary factors, including environment stimulus, individual organism, and response. The Flow theory provided further psychological state of people’s experiences, which helped to gain more insight to understand personal internal state. Based on these two approaches, our research intended to discuss how flow experience can lead to TikTok addiction behavior in adolescents and we also exhibited the analysis of potential antecedents of flow experience. The proposed model is shown in Figure 1.

### Environmental stimulus and adolescents’ flow experience

#### Information quality and flow experience

Information quality referred to the user’s evaluation of the immediacy, richness, and personalization of the content provided by media platforms (Jung et al., 2009), and previous studies confirmed the effect of information quality on users’ flow experience under the context of mobile internet sites (Zhou, 2014).

TikTok developed advanced algorithms to predict the information users were interested in by mining their personal information, thus continuously spoon-feeding videos to them, with occasional video type switches, so that the users can concentrate on the usage of the application and enjoyment, even lose track of time (Zhao, 2021). Besides, the format of the TikTok video was concise. Normally, it was only seconds to a few minutes. Compared to long videos and articles, short videos were more attractive since it fits users’ fast pace of life and fragmented time (Wu, 2020). Thus, people were not likely to stay focused on one message for a long period; however, they were easier to concentrate when one long piece of information was broken down into many short pieces (Karegar et al., 2020).

Thus, we hypothesize:

**Hypothesis 1:** Information quality has a positive influence on enjoyment.

**Hypothesis 2:** Information quality has a positive influence on concentration.

**Hypothesis 3:** Information quality has a positive influence on time distortion.

#### System quality and flow experience

System quality referred to the reliability, response time, and usability of the system (Gao and Bai, 2014). The research on flow experience confirmed that systems that run smoothly and are easy to use were more likely to attract customers (Aladwani and Palvia, 2002). For example, the research on online communication technology found that the ease of use significantly affected the flow experience (Chang and Wang, 2008).

In the context of TikTok, it had a simple operating system. The interaction was designed for immersive experiences and aimed to keep users in an extremely passive state to accept the recommended videos. Users only needed to swipe up the screen with low effort to glance at short videos, therefore, indulging TikTok and extending the usage time unconsciously (Zhao, 2021). Besides, the convergence of functions (such as integrated music, video, social, etc.) made TikTok more attractive. Users are swallowed by the fun of watching or editing short videos and even lost track of time (Liang, 2021).

Thus, we hypothesize:

**Hypothesis 4:** System quality has a positive influence on enjoyment.

**Hypothesis 5:** System quality has a positive influence on concentration.

**Hypothesis 6:** System quality has a positive influence on time distortion.

### Adolescents’ flow experience and TikTok addiction behavior

When the user was immersed in an activity, the flow experience was regarded as an emotional state, arousing the user’s curiosity (Webster et al., 1993). When users have a good experience, the flow experience is presented as a sense of pleasure, and users tend to spend more time participating in the activity (Bilgihan et al., 2014). For example, the more time consumers spent browsing the web, the more familiar they became with online retailers and the goods and services, which in turn promoted trust (Gulati and Sytch, 2008; Bilgihan et al., 2014). Besides, pleasure and the sense of belonging from social media were also found to trigger psychological processes of positive reinforcement and contribute to addiction behaviors later (Turel and Serenko, 2012).

Thus, the flow was regarded as a significant indicator of addiction behavior. Adolescents have the features of strong curiosity, however, they also lacked the ability to behavior control (Meeus et al., 2019; Lee and Baek, 2020). Therefore, when adolescents were absorbed into a flow state, they were easier to gain harmful habits, such as social media addiction (Zhao and Zhou, 2021), and online game addiction (Hu et al., 2019).

Thus, we hypothesize:

**Hypothesis 7:** Enjoyment has a positive influence on TikTok addiction behavior.

**Hypothesis 8:** Concentration has a positive influence on TikTok addiction behavior.

**Hypothesis 9:** Time distortion has a positive influence on TikTok addiction behavior.

Besides, we also considered that the flow experience mediates the relationship between information quality, system quality, and TikTok addiction behavior. This prediction originated from a previous study.

The user’s enjoyment of the experience could be improved by the high quality of the information and the system, and they might continue to be immersed in the whole experience (Heeter, 1992). Later, Pelet et al. (2017) found a positive relationship between information quality, system quality, and enjoyment. Park and Hwang (2009) confirmed that the flow mediates the relationship between telepresence and online gaming addiction. TikTok achieved personalization by customizing specific features and content for users. Studies showed that users’ participation in the personalization process could help them establish a sense of relevance with media platforms (Thongpapanl and Ashraf, 2011). Therefore, platforms that provided personalized services could in turn induce functional attachment between users and providers (Wan et al., 2017).

Thus, TikTok provided users with highly personalized videos and funny video editing functions that could potentially increase users’ enjoyment, and we hypothesize:

**Hypothesis 10:** Enjoyment mediates the relationship between information quality and TikTok addiction behavior.

**Hypothesis 11:** Enjoyment mediates the relationship between system quality and TikTok addiction behavior.

Likewise, concentration was proved to be a critical stage before forming addiction behavior (Khang et al., 2013). The high quality of information and system contributed to users’ heavy concentration on ongoing activities (Yang and Lee, 2018). In other words, when users interacted with entertaining media, they could have a strong interest and want to explore further, which had been confirmed by Wan and Chiou (2006).

In the context of TikTok, users could determine the values of the video and could use the platform easily. The interesting content and interactive system were very easy to be used as an external stimulus for teenagers to produce a concentrated use experience, which may also mediate the subsequent addiction behavior. In other words, when users were concentrating on the high quality of the information system, the addiction behavior was gradually formed.

Thus, we hypothesize:

**Hypothesis 12:** Concentration mediates the relationship between information quality and TikTok addiction behavior.

**Hypothesis 13:** Concentration mediates the relationship between system quality and TikTok addiction behavior.

Besides, because of the enjoyment they experienced while using TikTok, adolescents tend to immerse themselves in short videos for long periods, resulting in impaired attention mechanisms, and their arousal mechanisms may distort their perception of the time spent (Wittmann and Paulus, 2008). Adolescents might continuously interact with TikTok because of the attractive videos and finally gain addiction behavior. It was very easy for adolescents to immerse themselves in interesting content. They were constantly engaged, which led to the feeling of time distortion (Wan and Chiou, 2006). In other words, when the stimulus of the external environment was more intense, they could experience more pleasure, and the attention mechanism could be more severely damaged. Therefore, time distortion might mediate external stimulus and addiction behavior.

Thus, we hypothesize:

**Hypothesis 14:** Time distortion mediates the relationship between information quality and TikTok addiction behavior.

**Hypothesis 15:** Time distortion mediates the relationship between system quality and TikTok addiction behavior.

## Research methodology

### Research design and construct measurements

This study employed a quantitative approach paradigm for the primary data collection and analysis. An extensive review of previous literature enabled extractions of the measurement scale which were applied to develop a self-report survey form. The survey form was designed in English and Chinese. Since this survey was conducted in China, we applied a forward–backward translation method to ensure translation accuracy.

The items of each construct were taken from previous literature and applied to the TikTok addiction behavior context. Five-point Likert-type scales were used to measure each item from (1) “Strongly disagree” to (5) “Strongly agree.” Information quality was measured in three dimensions with 12 items from Zhang et al. (2016) and Lee and Kim (2017). System quality was measured in four dimensions with 14 items from Nelson et al. (2005). The Flow had three dimensions (enjoyment, concentration, and time distortion) with 6 (Cao et al., 2020), 4 (Chen et al., 2017), and 3 (Novak et al., 2000) items, respectively. TikTok addiction behavior was measured by 20 items from (Yu and Fu-min, 2005).

### Sample and data collection

This study conducted an online survey among TikTok (Douyin) users in China. Adolescents aged from 10 to 19 were recruited for this research. We first used a random sampling method to increase sample diversity. Gender, age, and education were taken into consideration when recruiting the first seed. After that, the virtual snowball sampling procedure was mainly used to recruit participants. All the participants were free to join and withdraw from the survey at their discretion.

Hair et al. (2017) recommended using G*Power to calculate the sample size for structural equation modeling, which was agreed by Ramayah et al. (2018). Our model had 5 predictors, therefore, to reach a medium effect size and a power of 0.8, a 90 minimum sample was needed. However, we expanded our sample size, since both Krejcie and Morgan (1970) and Hair et al. (2019) suggested to recruited around 384 respondents when the overall population size exceeds 100,000 to reduce heterogeneous populace issues. A recent study also recommended increasing the sample size to avoid online survey bias (Kirchherr and Charles, 2018). At last, 735 questionnaires were collected and 76 labeled as invalid were removed, 659 remained. The details of respondents’ demographic profiles are presented in Table 1.

**TABLE 1:** Respondents’ demographic profiles (*n* = 659).

Demographic factors	Frequency	Percentage (%)
**Gender**		
Male	289	43.9
Female	370	56.1
**Age**		
10–11	110	16.71
12–14	191	28.9
15–17	280	42.51
18–19	78	11.88
**Current education level**		
Did not attend school	0	0
Primary school	162	24.42
Secondary school	266	40.32
High school	175	26.60
Diploma	39	5.96
Bachelor	17	2.52
Master	0	0
Ph.D.	0	0

All the eligible respondents in this study were Chinese TikTok (Douyin) users and focused on adolescents aged 10–19. The data was collected from January 2022 to April 2022, when the COVID-19 epidemic unfolded a new wave of outbreaks. As shown in Table 1, 43.9% of the participants were male and 56.1% female. Most of the respondents (42.51%) were aged from 15 to 17 years, followed by 12 to 14 years (28.9%). All the participants were students and most of them were receiving secondary school level education in China.

### Common method bias

The common method bias was tested with the marker variable technique using the Partial Least Square (PLS) algorithm. This method was suggested since it examined the method variance when conducting statistical analysis (Lindell and Whitney, 2001; Podsakoff et al., 2003). Findings indicated that after adding the marker variable in the research model, the *R*^2^ value in TikTok addiction behavior changed slightly (from 0.252 to 0.253), less than 10%. These results confirmed that common method variance was not an issue in the data set (Lindell and Whitney, 2001). Table 2 presents the marker variables results.

**TABLE 2:** Comparison of *R*^2^ value between baseline model and marker included the model.

Relationships	Without marker variable	With marker variable
Concentration	0.244	0.244
Enjoyment	0.537	0.540
Time distortion	0.132	0.136
TikTok addiction	0.252	0.253

## Data analysis and results

Smart PLS was recommended by Hair et al. (2021) and Sarstedt et al. (2022) to test the research model, and scholars agreed that PLS-SEM is suitable for this study since it can estimate the measurement and structural model at the same time (Gefen et al., 2011). In line with previous interdisciplinary studies such as tourism management (Yang et al., 2021, 2022a), social media (Dalvi-Esfahani et al., 2021), consumer behavior (Yang et al., 2022b), and this study adopted Smart PLS 3.3.7 to conduct a two-stage approach to test model (Anderson and Gerbing, 1988).

### Measurement model

The internal consistency reliability was evaluated by Cronbach’s alpha and composite reliability. The results below exhibited that the model had sufficient internal consistency, since the values of Cronbach’s alpha and composite reliability were above 0.7 (Henseler et al., 2009; Hair et al., 2017). Next, the indicator reliability was good (outer loadings >0.6) (Chin, 1998) and the convergent validity was established (average variance extracted >0.5) (Fornell and Larcker, 1981; Hair et al., 2019). The details are presented in Table 3.

**TABLE 3:** Results summary for reflective measurement models.

Multi-dimensional constructs									

		Indicator reliability			Convergent validity	Constructs			Convergent validity
Constructs	Items		Internal consistency reliability				Internal consistency reliability		
							
First-order		Outer loadings	Cronbach’s alpha	Composite reliability	Average variance extracted	Second-order	Cronbach’s alpha	Composite reliability	Average variance extracted
							
		>0.60	>0.7	>0.7	>0.5		>0.7	>0.7	>0.5
Conciseness	IQC1	0.795	0.739	0.849	0.653	Information quality	0.901	0.917	0.668
	IQC2	0.825							
	IQC3	0.804							
Subscription	IQS1	0.782	0.838	0.892	0.675				
	IQS2	0.869							
	IQS3	0.864							
	IQS4	0.766							
Usefulness	IQU1	0.787	0.886	0.916	0.686				
	IQU2	0.838							
	IQU3	0.844							
	IQU4	0.823							
	IQU5	0.849							
Flexibility	SQF1	0.906	0.897	0.936	0.829	System quality	0.950	0.955	0.774
	SQF2	0.925							
	SQF3	0.900							
Integration	SQI1	0.917	0.913	0.945	0.852				
	SQI2	0.939							
	SQI3	0.913							
Ease of use	SQEF1	0.855	0.886	0.917	0.688				
	SQEF2	0.851							
	SQEF3	0.861							
	SQEF4	0.839							
	SQEF5	0.733							
Response time	SQRT1	0.874	0.863	0.916	0.785				
	SQRT2	0.895							
	SQRT3	0.889							

**Uni-dimensional constructs**					

**Constructs**	**Items**	**Indicator reliability**	**Internal consistency reliability**		**Convergent validity**
				
		**Outer loadings**	**Cronbach’s alpha**	**Composite reliability**	**Average variance extracted**
		**>0.60**	**>0.7**	**>0.7**	**>0.5**

Enjoyment	FE1	0.876	0.939	0.952	0.768
	FE2	0.916			
	FE3	0.916			
	FE4	0.904			
	FE5	0.791			
	FE6	0.846			
Concentration	FC1	0.904	0.931	0.951	0.829
	FC2	0.934			
	FC3	0.919			
	FC4	0.884			
Time distortion	FTD1	0.903	0.904	0.940	0.839
	FTD2	0.933			
	FTD3	0.912			
TikTok addiction behavior	TAB1/TAB11	0.722/0.818	0.969	0.971	0.623
	TAB2/TAB12	0.774/0.821			
	TAB3/TAB13	0.788/0.761			
	TAB4/TAB14	0.823/0.740			
	TAB5/TAB15	0.673/0.699			
	TAB6/TAB16	0.815/0.765			
	TAB7/TAB17	0.889/0.784			
	TAB8/TAB18	0.875/0.738			
	TAB9/TAB19	0.863/0.763			
	TAB10/TAB20	0.814/0.828			

IQC, conciseness; IQS, subscription; IQU, usefulness; SQF, flexibility; SQI, integration; SQEF, ease of use; SQRT, response time; FE, enjoyment; FC, concentration; FTD, time distortion; TAB, TikTok addiction behavior.

Next, we adopted the Heterotrait Monotrait (HTMT) technique to test the discriminant validity (Henseler et al., 2015). The results presented that all the constructs did not violate HTMT_0.85_, which confirmed that the discriminant validity was established (Henseler et al., 2015). Table 4 shows the findings that meet the threshold.

**TABLE 4:** Discriminant validity: Heterotrait Monotrait (HTMT) criterion.

	FC	FE	FTD	IQC	IQS	IQU	SQEF	SQF	SQI	SQRT	TAB
FC											
FE	0.728										
FTD	0.770	0.569									
IQC	0.347	0.436	0.333								
IQS	0.342	0.512	0.269	0.642							
IQU	0.465	0.701	0.299	0.599	0.734						
SQEF	0.529	0.756	0.423	0.495	0.576	0.725					
SQF	0.462	0.677	0.319	0.509	0.566	0.728	0.803				
SQI	0.379	0.626	0.280	0.425	0.480	0.718	0.813	0.785			
SQRT	0.463	0.676	0.335	0.484	0.542	0.679	0.858	0.764	0.697		
TAB	0.455	0.246	0.466	0.163	0.112	0.174	0.164	0.151	0.135	0.112	

FC, concentration; FE, enjoyment; FTD, time distortion; IQC, conciseness; IQS, subscription; IQU, usefulness; SQEF, ease of use; SQF, flexibility; SQI, integration; SQRT, response time; TAB, TikTok addiction behavior.

### Structural model

#### Hypothesis testing

In line with Hair et al. (2021), the path relationships were examined with 1,000 bootstrap samples and a one-tailed test with a 0.01 significance level. The PLS-bootstrapping results are presented in Tables 5, 6.

**TABLE 5:** Direct effect hypotheses.

Hypothesis					Bootstrapped		
					CI	BC	
					
Variable relationship	Path coefficient beta (β)	SD	*T*-statistics	*P*-values	1% LL	99% UL	Decision
Information quality → concentration	0.166	0.052	3.163	0.001	0.00	0.289	Accept
Information quality → enjoyment	0.225	0.039	5.694	0.000	0.129	0.330	Accept
Information quality → time distortion	0.116	0.058	1.998	0.023	−0.035	0.259	Reject
System quality → concentration	0.363	0.056	6.446	0.000	0.228	0.488	Accept
System quality → enjoyment	0.557	0.040	13.899	0.000	0.456	0.643	Accept
System quality → time distortion	0.271	0.059	4.561	0.000	0.131	0.401	Accept
Concentration → TikTok addiction behavior	0.364	0.061	5.923	0.000	0.245	0.513	Accept
Enjoyment → TikTok addiction behavior	−0.133	0.052	2.534	0.006	−0.223	−0.030	Accept
Time distortion → TikTok addiction behavior	0.263	0.056	4.685	0.000	0.130	0.371	Accept

**TABLE 6:** Summary of mediation test effects.

Hypothesis					Bootstrapped		
					CI	BC	
					
Variable relationship	Path coefficient beta (β)	SD	*T*-statistics	*P*-values	1% LL	99% UL	Decision
Information quality → enjoyment → TikTok addiction behavior	−0.030	0.012	2.447	0.007	−0.062	−0.003	Accept
Information quality → concentration → TikTok addiction behavior	0.060	0.022	2.769	0.003	0.012	0.116	Accept
Information quality → time distortion → TikTok addiction behavior	0.031	0.017	1.771	0.038	−0.005	0.081	Reject
System quality → enjoyment → TikTok addiction behavior	−0.074	0.031	2.400	0.008	−0.153	−0.006	Accept
System quality → concentration → TikTok addiction behavior	0.132	0.033	4.066	0.000	0.064	0.217	Accept
System quality → time distortion → TikTok addiction behavior	0.071	0.022	3.206	0.001	0.026	0.128	Accept

Our results showed that Information Quality had a significant influence on Enjoyment (β = 0.225, *t*-value = 5.694, *p* < 0.01) and Concentration (β = 0.166, *t*-value = 3.163, *p* < 0.01), thus supporting Hypothesis 1 and Hypothesis 2. However, the relationship between Information Quality and Time Distortion was insignificant (β = 0.116, *t*-value = 1.998, *p* > 0.01), therefore Hypothesis 3 was rejected. Besides, Hypothesis 4, Hypothesis 5, and Hypothesis 6 were supported, since System Quality exhibited positively influence on Enjoyment (β = 0.557, *t*-value = 13.899, *p* < 0.01), Concentration (β = 0.363, *t*-value = 6.446, *p* < 0.01), and Time Distortion (β = 0.271, *t*-value = 4.561, *p* < 0.01). And Enjoyment on TikTok Addiction Behavior Hypothesis 7 (β = −0.133, *t*-value = 2.534, *p* < 0.01), Concentration on TikTok Addiction Behavior Hypothesis 8 (β = 0.364, *t*-value = 5.923, *p* < 0.01), and Time Distortion on TikTok Addiction Behavior Hypothesis 9 (β = 0.263, *t*-value = 4.685, *p* < 0.01) were all statistically significant.

The mediating effect of Enjoyment (Information Quality → Enjoyment → TikTok Addiction Behavior: β = −0.030, *t*-value = 2.447, *p* < 0.01; System Quality → Enjoyment → TikTok Addiction Behavior: β = −0.074, *t*-value = 2.400, *p* < 0.01) and Concentration (Information Quality → Concentration → TikTok Addiction Behavior: β = 0.060, *t*-value = 2.769, *p* < 0.01; System Quality → Concentration → TikTok Addiction Behavior: β = 0.132, *t*-value = 4.066, *p* < 0.01) were significant. Time Distortion could mediate the relationship between System Quality and TikTok Addiction Behavior (β = 0.071, *t*-value = 3.206, *p* < 0.01), but have no significant mediation effect on Information Quality and TikTok Addiction Behavior (β = 0.031, *t*-value = 1.771, *p* > 0.01). Therefore, Hypothesis 10, Hypothesis 11, Hypothesis 12, Hypothesis 13, and Hypothesis 15 were all supported, while Hypothesis 14 was rejected.

#### Coefficient of determination (*R*^2^) and predictive relevance (*Q*^2^)

Besides, to justify the overall quality of the model, Hair et al. (2021) suggested evaluating the coefficient of determination (*R*^2^) and predictive relevance (*Q*^2^). Our results indicated that Enjoyment, Concentration, and Time Distortion accounted for a 25.2% variance in TikTok addiction behavior. Therefore, our model had satisfactory explanatory power. The predictive relevance between exogenous and endogenous variables also showed that the *Q*^2^ value of the construct was greater than zero (i.e., *Q*^2^ TikTok Addiction Behavior = 0.150). Table 7 shows the Model results for *R*^2^ and *Q*^2^.

**TABLE 7:** Model results for *R*^2^ and *Q*^2^.

Dependent Variables	*R* ^2^	Q^2^
Concentration	0.244	0.198
Enjoyment	0.537	0.409
Time distortion	0.132	0.107
TikTok addiction behavior	0.252	0.150

### Importance–performance matrix analysis

**FIGURE 2:**
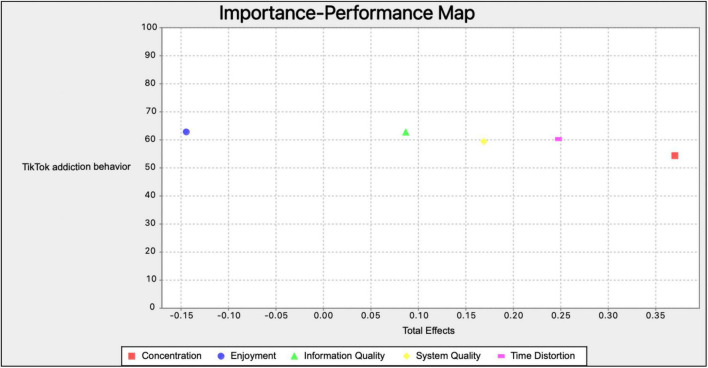
Importance–performance map of TikTok addiction behavior.

Hair et al. (2017) suggested testing importance–performance matrix analysis (IPMA) of latent variables scores to further examine its importance and performance. Table 8 and Figure 2 present the results of IPMA which targeted TikTok Addiction Behavior. Results showed that Concentration had the highest importance value (0.370), which was followed by Time Distortion (0.248) in influencing TikTok Addiction Behavior, while Enjoyment had the highest performance value (62.820) and the lowest importance (−0.144).

**TABLE 8:** Importance–performance map (TikTok addiction behavior) (constructs, unstandardized effects).

Structural model	Importance (total effects)	Performance
Information quality	0.087	62.723
System quality	0.169	59.432
Enjoyment	−0.144	62.820
Concentration	0.370	54.364
Time distortion	0.248	60.259

## Discussion and conclusion

### Discussion

Based on the “Stimulus–Organism–Response” paradigm, this study attempted to investigate how the information system affected flow experience and further triggered adolescents’ addiction to TikTok. Some findings were of significance.

First, this study determined that information quality and system quality worked as stimulus factors that have partial positive influence on flow experience. This was aligned with former studies (Gao and Bai, 2014; Gao et al., 2015). Besides, the findings of this study indicated that the linkage between system quality and flow experience (enjoyment, concentration, and time distortion) were relatively higher than the relationships between information quality and all dimensions of flow experience, while the mediation effect of time distortion to information quality and TikTok addiction behavior was insignificant. In other words, the system quality contributed more to users’ flow experience and addiction behavior, because TikTok’s rigorous algorithm system enhanced the deep interaction with the user and therefore provided high-quality creative content (Yu, 2019), making the videos highly comprehensive and meeting user requirements (Zhang and Liu, 2021). Thereby adolescents were more likely to step into a flow experience.

Second, this study regarded the flow as an organism and broke it into enjoyment, concentration, and time distortion. We empirically proved their positive effect on TikTok addiction behavior. A previous study found that the flow directly influenced social media addiction (Lee et al., 2018), and this study further explained that the specific state of flow (enjoyment, concentration, and time distortion) mediated the effects of the TikTok environment on addiction behavior.

Third, we found that concentration was the most important factor in TikTok addiction behavior. The flow theory may provide a possible explanation. Wang (2020) and Pelet et al. (2017) agreed that concentration was the key factor in flow. When users, especially adolescents users, were having fun online, it was hard for them to break off from the ongoing entertainment activities, except for compelling interruption from the real world (Rau et al., 2006; Montag et al., 2019), for example, parental control, academic work pressure, etc. Therefore, concentration was the critical representation of the user’s deep immersion which led to TikTok addiction behavior.

### Conclusion

#### Theoretical implications

First, this study contributed to enriching the concept of environmental components on TikTok. Previous literature only proposed environment types based on platform design, website layout, and video content characteristics (Cao et al., 2020; Wang, 2020), while drawing much attention to the negative consequences of addiction behavior and how personal state or technical factors independently lead to addiction behavior (Hasan et al., 2018). This may hinder a comprehensive understanding of the online environment on TikTok. Our work extended the media literature by integrating information quality elements (such as conciseness, usefulness, and subscription) and system quality elements (flexibility, integration, ease of use, and response time) as predictors in the TikTok context, thus, we provided a new perspective on the relationship between the online environment of TikTok and the psychology of Chinese adolescents’ users. This study also contributed to the media literature by confirming the applicability of the S–O–R paradigm combined with flow theory in the context of TikTok: we conceptualized each category of the environment as stimulus (information quality and system quality), organism (enjoyment, concentration, and time distortion), response (addiction behavior), and generalized causality.

Second, there was very limited research on how to specify each component of flow effect on addiction behavior. While previous studies treated the flow as a wholistic construct (Chou and Ting, 2003; Kim et al., 2017; Brailovskaia and Teichert, 2020; Zhao and Zhou, 2021), we empirically examined the flow in three specific components and found concentration was the most important factor to affect TikTok addiction behavior. Our findings pointed out that the flow, as the best experience, has an important effect on TikTok addiction behavior. We demonstrated the significant mediating effect of the three parts of flow (enjoyment, concentration, and time distortion) to link the information system to TikTok addiction behavior. Thus, our study gained useful insight into understanding the causes and consequences of flow experience in the context of TikTok.

Third, the information quality and system quality working as antecedents of flow theory had been applied to social networking services (Gao and Bai, 2014), but when applied to TikTok, our results were different. Gao and Bai (2014) found that information quality can significantly influence flow, while the relationship between system quality and flow was not identified. However, our results showed information quality positively influenced enjoyment and concentration but did not have an effect on time distortion, while system quality was positively related to all of them. These results were partially contradictory to Gao and Bai’s (2014) study. Therefore, we empirically revised the flow theory under the environment of TikTok.

The results could be explained from two aspects. First, Gao and Bai (2014) only roughly studied the influence of information and system on the flow, but this study specifically refined each part of the flow, so the results were different. Besides, the research of Gao and Bai (2014) was applied in the context of social network services 8 years ago. TikTok system was equipped with more advanced algorithm technology, which was more intelligent than previous media platforms. It can recommend content in a more accurate and personalized way by using a hierarchical interest tag tree, user role, and partitioned data bucket strategy (Zhao, 2021). After identifying the basic information and viewing preferences of adolescents, the algorithm can recommend more entertaining videos to them and provide timely news about the epidemic during the period of COVID-19. Therefore, when viewing these videos, adolescents felt enjoyment and devoted more concentration due to the entertainment and topic sensitivity characteristics.

However, under the 9-year compulsory education and proper parental guidance, Chinese adolescents had the ability to evaluate video information. After realizing the lack of meaning and false information in the short videos, they might feel nervous and guilty about using TikTok continuously due to a lack of self-control, so it was hard to assess the time distortion. This phenomenon had been demonstrated in previous studies (Luqman et al., 2017, 2020).

#### Practical implications

This study also had some practical implications. First, TikTok developers should beware that aside from gaining benefits to the enterprise, TikTok has a need to contribute to society. Attracting users to become addicted to TikTok is not a positive phenomenon. TikTok should be constructed into a platform that can serve as both entertainment and information delivery. Based on our findings, concentration was the most important factor leading to TikTok addiction behavior, therefore, algorithms and systems should be optimized to design an effective function that can interrupt users who have been immersed in TikTok for an excessive amount of time or recommend short educational videos to cultivate users to form healthy TikTok usage habits.

Adolescents may not voluntarily reduce their overuse of TikTok, since they are still forming a psychological mechanism process, are curious about their surroundings, and usually lack self-control. Therefore, healthy and positive psychological and behavioral habits should be cultivated, and behavior cognitive and critical thinking abilities should be fostered to increase adolescents’ ability to control themselves.

However, self-control is not an easy task. Thus, from the perspective of school and parents, it would be helpful for schools to provide adolescents with more valuable and engaging online activities to decrease time spent on TikTok. Besides, parental control had proven as an effective way to avoid and alleviate adolescents’ problems of social media addiction (Lee and Ogbolu, 2018). Thus, parents should intervene in adolescents’ TikTok use and break adolescents’ concentration.

#### Limitations and future research

This study had some limitations that provide insights for further research. First, our sample only contained 659 Chinese adolescents, this may not reflect other larger geographical and cultural areas. Future studies can consider cultural differences, conduct cross-cultural studies, or expand the age range of samples to detect differences between age groups. In addition, there are other short video applications, including Kuaishou, etc. Depending on the type of application, there may be biases associated with user behavior or addiction behavior. Subsequent work can use multi-group analysis to compare the behavior of Chinese users using short video media. Last, our results confirmed that enjoyment, concentration, and time distortion explained the 25.2% variance in TikTok addiction behavior. Thus, future studies could introduce new variables such as habit and engagement to increase model explanatory ability.

## Data availability statement

The raw data supporting the conclusions of this article will be made available by the authors, without undue reservation.

## Ethics statement

Ethical review and approval was not required for the study on human participants in accordance with the local legislation and institutional requirements. Written informed consent from the patients/participants or patients/participants legal guardian/next of kin was not required to participate in this study in accordance with the national legislation and the institutional requirements.

## Author contributions

YQ and BO: conceptualization and writing – literature review. YQ: writing—original and proofreading, data collection, research methodology, data analysis, editing, and formatting. YQ, BO, and AM: reviewing and revising. BO: supervision and project administration. All authors contributed to the article and approved the submitted version.

## References

[B1] Aladwani A. M. Palvia P. C. (2002). Developing and validating an instrument for measuring user-perceived web quality. *Inform. Manag.* 39 467–476. 10.1016/S0378-7206(01)00113-6

[B2] Al-Fraihat D. Joy M. Masa’deh R. Sinclair J. (2020). Evaluating E-learning systems success: an empirical study. *Comp. Human Behav.* 102 67–86. 10.1016/j.chb.2019.08.004

[B3] Anderson J. C. Gerbing D. W. (1988). Structural equation modeling in practice: a review and recommended two-step approach. *Psychol. Bull.* 103 411–423. 10.1037/0033-2909.103.3.411

[B4] Bagozzi R. P. (1986). Attitude formation under the theory of reasoned action and a purposeful behaviour reformulation. *Br. J. Soc. Psychol.* 25 95–107. 10.1111/j.2044-8309.1986.tb00708

[B5] Bilgihan A. Okumus F. Nusair K. Bujisic M. (2014). Online experiences: flow theory, measuring online customer experience in e-commerce and managerial implications for the lodging industry. *Inform. Technol. Tour.* 14 49–71. 10.1007/s40558-013-0003-3

[B6] Brailovskaia J. Teichert T. (2020). “I like it” and “I need it”: relationship between implicit associations, flow, and addictive social media use. *Comp. Hum. Behav.* 113:106509. 10.1016/j.chb.2020.106509

[B7] Cao X. Gong M. Yu L. Dai B. (2020). Exploring the mechanism of social media addiction: an empirical study from WeChat users. *Internet Res.* 30 1305–1328. 10.1108/INTR-08-2019-0347

[B8] Cao X. Sun J. (2018). Exploring the effect of overload on the discontinuous intention of social media users: an S-O-R perspective. *Comp. Hum. Behav.* 81 10–18. 10.1016/j.chb.2017.11.035

[B10] Casale S. Rugai L. Fioravanti G. (2018). Exploring the role of positive metacognitions in explaining the association between the fear of missing out and social media addiction. *Addict. Behav.* 85 83–87. 10.1016/j.addbeh.2018.05.020 29864680

[B11] Chang H. H. Wang I. C. (2008). An investigation of user communication behavior in computer mediated environments. *Comp. Hum. Behav.* 24 2336–2356. 10.1016/j.chb.2008.01.001

[B12] Chen C. Zhang K. Z. K. Gong X. Zhao S. J. Lee M. K. O. Liang L. (2017). Understanding compulsive smartphone use: an empirical test of a flow-based model. *Int. J. Inform. Manag.* 37 438–454. 10.1016/j.ijinfomgt.2017.04.009

[B13] Cheng Y. M. (2019). A hybrid model for exploring the antecedents of cloud ERP continuance: roles of quality determinants and task-technology fit. *Int. J. Web Inform. Syst.* 15 215–235. 10.1108/IJWIS-07-2018-0056

[B14] Chin W. W. (1998). Issues and opinion on structural equation modeling. *MIS Q.* 22 VII–XVI.

[B15] Chopdar P. K. Balakrishnan J. (2020). Consumers response towards mobile commerce applications: S-O-R approach. *Int. J. Inform. Manag.* 53:102106. 10.1016/j.ijinfomgt.2020.102106

[B16] Chou T. J. Ting C. C. (2003). The role of flow experience in cyber-game addiction. *Cyberpsychol. Behav.* 6 663–675. 10.1089/109493103322725469 14756934

[B17] Csikszentmihalyi M. (1975). Play and intrinsic rewards. *J. Human. Psychol.* 15 41–63. 10.1177/002216787501500306

[B18] Dalvi-Esfahani M. Niknafs A. Alaedini Z. Barati Ahmadabadi H. Kuss D. J. Ramayah T. (2021). Social media addiction and empathy: moderating impact of personality traits among high school students. *Telematics Inform.* 57:101516. 10.1016/j.tele.2020.101516

[B19] DeLone W. H. McLean E. R. (2003). The delone and mclean model of information systems success: a ten-year update. *J. Manag. Inform. Syst.* 19 9–30. 10.1080/07421222.2003.11045748

[B20] Ding D. X. Hu P. J. H. Verma R. Wardell D. G. (2010). The impact of service system design and flow experience on customer satisfaction in online financial services. *J. Service Res.* 13 96–110. 10.1177/1094670509350674

[B21] Donovan R. J. Rossiter J. R. (1982). Store atmosphere-an environmental psychology approach. *J. Retailing* 58 34–57.

[B22] D’souza L. (2019). Instagram addiction among students pursuing medical and dental courses: a comparative study. *Int. J. Indian Psychol.* 7 443–448. 10.25215/0701.049

[B23] Du X. Liechty T. Santos C. A. Park J. (2020). “I want to record and share my wonderful journey”: Chinese Millennials’ production and sharing of short-form travel videos on TikTok or Douyin. *Curr. Issues Tour.* 1–13. 10.1080/13683500.2020.1810212 [Epub ahead of print].

[B24] Fornell C. G. Larcker D. F. (1981). Evaluating structural equation models with unobservable variables and measurement error. *J. Market. Res.* 18 39–50. 10.1177/002224378101800104

[B25] Gao L. Bai X. (2014). An empirical study on continuance intention of mobile social networking services: integrating the IS success model, network externalities and flow theory. *Asia Pacific J. Market. Logistics* 26 168–189. 10.1108/APJML-07-2013-0086

[B26] Gao L. Waechter K. A. Bai X. (2015). Understanding consumers’ continuance intention towards mobile purchase: a theoretical framework and empirical study - a case of China. *Comp. Hum. Behav.* 53 249–262. 10.1016/j.chb.2015.07.014

[B27] Gao W. Liu Z. Li J. (2017). How does social presence influence SNS addiction? a belongingness theory perspective. *Comp. Hum. Behav.* 77 347–355. 10.1016/j.chb.2017.09.002

[B28] Gao L. Bai X. Park A. (Tony) (2017). Understanding sustained participation in virtual travel communities from the perspectives of is success model and flow theory. *J. Hosp. Tour. Res.* 41 475–509. 10.1177/1096348014563397

[B29] Gefen D. Rigdon E. E. Straub D. (2011). Editor’s comments: an update and extension to SEM guidelines for administrative and social science. *Source MIS Q.* 35 iii–xiv. 10.2307/23044042

[B30] Gulati R. Sytch M. (2008). Does familiarity breed trust? revisiting the antecedents of trust. *Manag. Decis. Econ.* 29 165–190. 10.1002/mde.1396

[B31] Hair J. F. Jr. Matthews L. M. Matthews R. L. Sarstedt M. (2017). PLS-SEM or CB-SEM: updated guidelines on which method to use. *Int. J. Multivariate Data Anal.* 1:107. 10.1504/ijmda.2017.10008574 35009967

[B32] Hair J. F. Hult G. T. M. Ringle C. Sarstedt M. Danks N. Ray S. (2021). *Partial Least Squares Structural Equation Modeling (PLS-SEM) Using R: A Workbook.* Berlin: Springer. 10.1007/978-3-030-80519-7

[B33] Hair J. F. Risher J. J. Sarstedt M. Ringle C. M. (2019). When to use and how to report the results of PLS-SEM. *Eur. Bus. Rev.* 31 2–24. 10.1108/EBR-11-2018-0203

[B34] Hasan M. R. Jha A. K. Liu Y. (2018). Excessive use of online video streaming services: impact of recommender system use, psychological factors, and motives. *Comp. Hum. Behav.* 80 220–228. 10.1016/j.chb.2017.11.020

[B35] Heeter C. (1992). Being there: the subjective experience of presence. *Presence Teleoperators Virtual Environ.* 1 262–271. 10.1162/pres.1992.1.2.262

[B36] Henseler J. Ringle C. M. Sarstedt M. (2015). A new criterion for assessing discriminant validity in variance-based structural equation modeling. *J. Acad. Market. Sci.* 43 115–135. 10.1007/s11747-014-0403-8

[B37] Henseler J. Ringle C. M. Sinkovics R. R. (2009). The use of partial least squares path modeling in international marketing. *Adv. Int. Market.* 20 277–319. 10.2196/jmir.3122 24918859 PMC4071227

[B38] Hoffman D. L. Novak T. P. (2009). Flow online: lessons learned and future prospects. *J. Interact. Market.* 23 23–34. 10.1016/j.intmar.2008.10.003

[B39] Holbrook M. B. Hirschman E. C. (1982). Experiential aspects of consumption: consumer fantasies, feelings, and fun. *J. Consum. Res.* 9 132–140. 10.1086/208906

[B40] Hu E. Stavropoulos V. Anderson A. Scerri M. Collard J. (2019). Internet gaming disorder: feeling the flow of social games. *Add. Behav. Rep.* 9:100140. 10.1016/j.abrep.2018.10.004 31193693 PMC6541905

[B41] Iram Aggarwal H. (2020). Time series analysis of pubg and tiktok applications using sentiments obtained from social media-twitter. *Adv. Math. Sci. J.* 9 4047–4057. 10.37418/amsj.9.6.86

[B42] Jung H. Zhou Q. (2019). Learning and sharing creative skills with short videos: a case study of user behavior in Tiktok and Bilibili. *Int. Assoc. Soc. Des. Res. Conference* 10 25–50.

[B43] Jung Y. Perez-Mira B. Wiley-Patton S. (2009). Consumer adoption of mobile TV: examining psychological flow and media content. *Comp. Hum. Behav.* 25 123–129. 10.1016/j.chb.2008.07.011

[B44] Karegar F. Pettersson J. S. Fischer-Hübner S. (2020). The dilemma of user engagement in privacy notices: effects of interaction modes and habituation on user attention. *ACM Trans. Privacy Security* 23 1–38. 10.1145/3372296

[B45] Khang H. Kim J. K. Kim Y. (2013). Self-traits and motivations as antecedents of digital media flow and addiction: the internet, mobile phones, and video games. *Comp. Hum. Behav.* 29 2416–2424. 10.1016/j.chb.2013.05.027

[B46] Kim M. J. Lee C. K. Bonn M. (2017). Obtaining a better understanding about travel-related purchase intentions among senior users of mobile social network sites. *Int. J. Inform. Manag.* 37 484–496. 10.1016/j.ijinfomgt.2017.04.006

[B47] Kim M. J. Lee C. K. Jung T. (2020). Exploring consumer behavior in virtual reality tourism using an extended stimulus-organism-response model. *J. Travel Res.* 59 69–89. 10.1177/0047287518818915

[B48] Kirchherr J. Charles K. (2018). Enhancing the sample diversity of snowball samples: recommendations from a research project on anti-dam movements in Southeast Asia. *PLoS One* 13:e0201710. 10.1371/journal.pone.0201710 30133457 PMC6104950

[B49] Klobas J. E. McGill T. J. Moghavvemi S. Paramanathan T. (2018). Compulsive YouTube usage: a comparison of use motivation and personality effects. *Comp. Hum. Behav.* 87 129–139. 10.1016/j.chb.2018.05.038

[B50] Krejcie V. R. Morgan W. D. (1970). Determining sample size for research activities. *Educ. Psychol. Meas.* 30 607–610. 10.1177/001316447003000308

[B51] Lee C. H. Chiang H. Sen Hsiao K. L. (2018). What drives stickiness in location-based AR games? an examination of flow and satisfaction. *Telematics Inform.* 35 1958–1970. 10.1016/j.tele.2018.06.008

[B52] Lee E. J. Ogbolu Y. (2018). Does parental control work with smartphone addiction?: a cross-sectional study of children in South Korea. *J. Add. Nursing* 29 128–138. 10.1097/JAN.0000000000000222 29864060

[B53] Lee H. W. Gipson C. Barnhill C. (2017). Experience of spectator flow and perceived stadium atmosphere: moderating role of team identification. *Sport Market. Q.* 26 87–98.

[B54] Lee S. Kim B. G. (2017). The impact of qualities of social network service on the continuance usage intention. *Manag. Decis.* 55 701–729. 10.1108/MD-10-2016-0731

[B55] Lee S. U. Baek H. (2020). Does parental intervention matter to diminish drinking behaviors among american adolescents? *Substance Use Misuse* 55 1300–1308. 10.1080/10826084.2020.1735440 32162996

[B56] Leong L. Y. Hew T. S. Ooi K. B. Lee V. H. Hew J. J. (2019). A hybrid SEM-neural network analysis of social media addiction. *Expert Syst. Appl.* 133 296–316. 10.1016/j.eswa.2019.05.024

[B57] Li Y. Shang H. (2020). Service quality, perceived value, and citizens’ continuous-use intention regarding e-government: empirical evidence from China. *Inform. Manag.* 57:103197. 10.1016/j.im.2019.103197

[B58] Liang L. Zhou D. Yuan C. Shao A. Bian Y. (2016). Gender differences in the relationship between internet addiction and depression. *Comput. Hum. Behav.* 63 463–470. 10.1016/j.chb.2016.04.043

[B59] Liang X. (2021). “Research on how to perceive their behavior for international high school students based on using TikTok with semi-structured interview,” in *Proceedings of the 2021 6th International Conference on Social Sciences and Economic Development (ICSSED 2021)*, (Amsterdam: Atlantis Press). 10.2991/assehr.k.210407.151

[B60] Lin J. Lin S. Turel O. Xu F. (2020). The buffering effect of flow experience on the relationship between overload and social media users’ discontinuance intentions. *Telematics Inform.* 49:101374. 10.1016/j.tele.2020.101374

[B61] Lindell M. K. Whitney D. J. (2001). Accounting for common method variance in cross-sectional research designs. *J. Appl. Psychol.* 86 114–121. 10.1037/0021-9010.86.1.114 11302223

[B62] Luqman A. Cao X. Ali A. Masood A. Yu L. (2017). Empirical investigation of Facebook discontinues usage intentions based on SOR paradigm. *Comp. Hum. Behav.* 70 544–555. 10.1016/j.chb.2017.01.020

[B63] Luqman A. Masood A. Weng Q. Ali A. Rasheed M. I. (2020). Linking excessive SNS use, technological friction, strain, and discontinuance: the moderating role of guilt. *Inform. Syst. Manag.* 37 94–112. 10.1080/10580530.2020.1732527

[B64] Meeus A. Eggermont S. Beullens K. (2019). Constantly connected: the role of parental mediation styles and self-regulation in pre-and early adolescents’ problematic mobile device use. *Hum. Commun. Res.* 45 119–147. 10.1093/hcr/hqy015

[B65] Mehrabian A. Russell J. A. (1974). Measure of arousal seeking tendency. *Environ. Behav.* 5 315–333. 10.1177/001391657300500303

[B66] Min Z. Jie Z. Xiao X. Mengyuan Q. Youhai L. Hui Z. (2020). How destination music affects tourists’ behaviors: travel with music in Lijiang, China. *Asia Pac. J. Tour. Res.* 25 131–144. 10.1080/10941665.2019.1683046

[B67] Montag C. Lachmann B. Herrlich M. Zweig K. (2019). Addictive features of social media/messenger platforms and freemium games against the background of psychological and economic theories. *Int. J. Environ. Res. Public Health* 16:2612. 10.3390/ijerph16142612 31340426 PMC6679162

[B68] Montag C. Yang H. Elhai J. D. (2021). On the psychology of tiktok use: a first glimpse from empirical findings. *Front. Public Health* 9:641673. 10.3389/fpubh.2021.641673 33816425 PMC8010681

[B69] Moon H. Yoon H. J. Han H. (2016). Role of airport physical environments in the satisfaction generation process: mediating the impact of traveler emotion. *Asia Pac. J. Tour. Res.* 21 193–211. 10.1080/10941665.2015.1048260

[B70] Mou J. B. (2020). Study on social media marketing campaign strategy-tiktok and instagram. *MIT Sloan School Manag.* 3 1–41.

[B71] Nelson R. R. Todd P. A. Wixom B. H. (2005). Antecedents of information and system quality: an empirical examination within the context of data warehousing. *J. Manag. Inform. Syst.* 21 199–235. 10.1080/07421222.2005.11045823

[B72] Novak T. P. Hoffman D. L. Yung Y. F. (2000). Measuring the customer experience in online environments: a structural modeling approach. *Market. Sci.* 19 22–42. 10.1287/mksc.19.1.22.15184 19642375

[B73] Park S. Hwang H. S. (2009). “Understanding online game addiction: connection between presence and flow,” in *Human-Computer Interaction. Interacting in Various Application Domains. HCI 2009. Lecture Notes in Computer Science*, ed. Jacko J. A. (Berlin: Springer). 10.1007/978-3-642-02583-9_42

[B74] Pelet J. É Ettis S. Cowart K. (2017). Optimal experience of flow enhanced by telepresence: evidence from social media use. *Inform. Manag.* 54 115–128. 10.1016/j.im.2016.05.001

[B75] Podsakoff P. M. MacKenzie S. B. Lee J. Y. Podsakoff N. P. (2003). Common method biases in behavioral research: a critical review of the literature and recommended remedies. *J. Appl. Psychol.* 88 879–903. 10.1037/0021-9010.88.5.879 14516251

[B76] Ramayah T. Cheah J. Chuah F. Memon H. T. Ali M. (2018). Partial least squares structural equation modeling with R. *Practical Assess. Res. Eval.* 21 1–16.

[B77] Rau P. L. P. Peng S. Y. Yang C. C. (2006). Time distortion for expert and novice online game players. *Cyberpsychol. Behav.* 9 396–403. 10.1089/cpb.2006.9.396 16901242

[B78] Russell J. A. Pratt G. (1980). A description of the affective quality attributed to environments. *J. Pers. Soc. Psychol.* 38:311. 10.1111/j.1365-2648.2008.04775.x 18990113

[B79] Sarstedt M. Hair J. F. Pick M. Liengaard B. D. Radomir L. Ringle C. M. (2022). Progress in partial least squares structural equation modeling use in marketing research in the last decade. *Psychol. Market.* 2021 1035–1064. 10.1002/mar.21640

[B80] Smith T. Short A. (2022). Needs affordance as a key factor in likelihood of problematic social media use: validation, latent Profile analysis and comparison of TikTok and Facebook problematic use measures. *Addict. Behav.* 129:107259. 10.1016/j.addbeh.2022.107259 35091200

[B81] Suh A. Prophet J. (2018). The state of immersive technology research: a literature analysis. *Comp. Hum. Behav.* 86 77–90. 10.1016/j.chb.2018.04.019

[B82] Suhartanto D. Helmi Ali M. Tan K. H. Sjahroeddin F. Kusdibyo L. (2017). Loyalty towards online food delivery service: the role of e-service quality and food quality. *J. Foodservice Bus. Res.* 22 1–17. 10.1080/15378020.2018.1546076

[B83] Sun J. Chen P. J. Ren L. Shih E. H. W. Ma C. Wang H. (2021). Place attachment to pseudo establishments: an application of the stimulus-organism-response paradigm to themed hotels. *J. Bus. Res.* 129 484–494. 10.1016/j.jbusres.2020.10.005

[B84] Sun Y. Q. Zhao Y. Jia S. Q. Zheng D. Y. (2015). “Understanding the antecedents of mobile game addiction: the roles of perceived visibility, perceived enjoyment and flow,” in *Proceedings of the Pacific Asia Conference on Information Systems, PACIS 2015 - Proceedings*, (Singapore: Marian Bay Sands).

[B85] Tarafdar M. Maier C. Laumer S. Weitzel T. (2020). Explaining the link between technostress and technology addiction for social networking sites: a study of distraction as a coping behavior. *Inform. Systems J.* 30 96–124. 10.1111/isj.12253

[B86] Teo T. S. H. Lim V. K. G. Lai R. Y. C. (1999). Intrinsic and extrinsic motivation in internet usage. *Omega* 27 25–37. 10.1016/S0305-0483(98)00028-0

[B87] Thongpapanl N. Ashraf A. R. (2011). Enhancing online performance through website content and personalization. *J. Comput. Inf. Syst.* 52 3–13.

[B88] Tian Y. Bian Y. Han P. Gao F. Wang P. (2017). Associations between psychosocial factors and generalized pathological internet use in Chinese university students: a longitudinal cross-lagged analysis. *Comp. Hum. Behav.* 72 178–188. 10.1016/j.chb.2017.02.048 30376647

[B89] Tuerlan T. Li S. Scott N. (2021). Customer emotion research in hospitality and tourism: conceptualization, measurements, antecedents and consequences. *Int. J. Contemp. Hosp. Manag.* 33 2741–2772. 10.1108/IJCHM-11-2020-1257

[B90] Turel O. Serenko A. (2012). The benefits and dangers of enjoyment with social networking websites. *Eur. J. Inf. Syst.* 21 512–528. 10.1057/ejis.2012.1

[B91] Wan C. S. Chiou W. B. (2006). Psychological motives and online games addiction: atest of flow theory and humanistic needs theory for taiwanese adolescents. *CyberPsychol. Behav.* 9 317–324. 10.1089/cpb.2006.9.317 16780399

[B92] Wan J. Lu Y. Wang B. Zhao L. (2017). How attachment influences users’ willingness to donate to content creators in social media: a socio-technical systems perspective. *Inf. Manag.* 54 837–850. 10.1016/j.im.2016.12.007

[B93] Wang Y. (2020). Humor and camera view on mobile short-form video apps influence user experience and technology-adoption intent, an example of TikTok (DouYin). *Comput. Hum. Behav.* 110:106373. 10.1016/j.chb.2020.106373

[B94] Webster J. Trevino L. K. Ryan L. (1993). The dimensionality and correlates of flow in human-computer interactions. *Comput. Hum. Behav.* 9 411–426. 10.1016/0747-5632(93)90032-N

[B95] Weimann G. Masri N. (2020). Research note: spreading hate on tiktok. *Stud. Conflict Terror.* 20, 1–14. 10.1080/1057610X.2020.1780027

[B96] Wittmann M. Paulus M. P. (2008). Decision making, impulsivity and time perception. *Trends Cogn. Sci.* 12 7–12. 10.1016/j.tics.2007.10.004 18042423

[B97] Wu L. (2020). Comparative analysis of video stories and user behaviors on wechat and Tik Tok. *Adv. Soc. Sci. Educ. Hum. Res.* 496 329–333. 10.2991/assehr.k.201214.518 32175718

[B98] Yang H. Lee H. (2018). Exploring user acceptance of streaming media devices: an extended perspective of flow theory. *Inf. Syst. E-Bus. Manag.* 16 1–27. 10.1007/s10257-017-0339-x

[B99] Yang S. Isa S. M. Ramayah T. (2022a). Does uncertainty avoidance moderate the effect of self-congruity on revisit intention? a two-city (Auckland and Glasgow) investigation. *J. Destination Mark. Manag.* 24:100703. 10.1016/j.jdmm.2022.100703

[B100] Yang S. Isa S. M. Wu H. Thurasamy R. Fang X. Fan Y. (2022b). Effects of stores’ environmental components on chinese consumers’ emotions and intentions to purchase luxury brands: integrating partial least squares-structural equation modeling and fuzzy-set qualitative comparative analysis approaches. *Front. Psychol.* 13:840413. 10.3389/fpsyg.2022.840413 35465550 PMC9029600

[B101] Yang S. Isa S. M. M. Ramayah T. Wen J. Goh E. (2021). Developing an extended model of self-congruity to predict Chinese tourists’ revisit intentions to New Zealand: the moderating role of gender. *Asia Pacific J. Mark. Logistics* 34 1459–1481. 10.1108/APJML-05-2021-0346

[B102] Yen N. Y. Chen C. C. Hu J. J. (2018). Investigating the customer’s intention in the “Clicks-and-Mortar” business model. *J. Ambient Intell. Humaniz. Comput.* 1–11. 10.1007/s12652-018-0929-6

[B103] Young K. S. (1999). Internet addiction: evaluation and treatment. *Bmj* 319:9910351. 10.1136/sbmj.9910351

[B104] Yu B. Fu-min F. (2005). A study on the internet dependence of college students: the revising and applying of a measurement. *Psychol. Dev. Educ.* 21 99–104.

[B105] Yu J. X. (2019). Research on TikTok APP based on user-centric theory. *Appl. Sci. Innov. Res.* 3:28. 10.22158/asir.v3n1p28

[B106] Zhang K. Min Q. Liu Z. Liu Z. (2016). Understanding microblog continuance usage intention: an integrated model. *Aslib J. Inf. Manag.* 68 772–792. 10.1108/AJIM-03-2016-0025

[B107] Zhang M. Liu Y. (2021). A commentary of TikTok recommendation algorithms in MIT technology review 2021. *Fundam. Res.* 1 846–847. 10.1016/j.fmre.2021.11.015

[B108] Zhang R. P. Bai B. Y. Jiang S. Yang S. Zhou Q. (2019). Parenting styles and internet addiction in Chinese adolescents: conscientiousness as a mediator and teacher support as a moderator. *Comput. Hum. Behav.* 101 144–150. 10.1016/j.chb.2019.07.019

[B109] Zhang X. Wu Y. Liu S. (2019). Exploring short-form video application addiction: socio-technical and attachment perspectives. *Telemat. Inform.* 42:101243. 10.1016/j.tele.2019.101243

[B110] Zhang Y. Qin X. Ren P. (2018). Adolescents’ academic engagement mediates the association between internet addiction and academic achievement: the moderating effect of classroom achievement norm. *Comput. Hum. Behav.* 89 299–307. 10.1016/j.chb.2018.08.018

[B111] Zhao N. Zhou G. (2021). COVID-19 stress and addictive social media use (SMU): mediating role of active use and social media flow. *Front. Psychiatry* 12:635546. 10.3389/fpsyt.2021.635546 33633616 PMC7899994

[B112] Zhao Y. Wang A. Sun Y. (2020). Technological environment, virtual experience, and MOOC continuance: a stimulus-organism-response perspective. *Comput. Educ.* 144:103721. 10.1016/j.compedu.2019.103721

[B113] Zhao Z. (2021). Analysis on the douyin (Tiktok) mania phenomenon based on recommendation algorithms. *E3S Web Conf.* 235:03029. 10.1051/e3sconf/202123503029

[B114] Zhou T. (2014). Understanding continuance usage intention of mobile internet sites. *Universal Access Inf. Soc.* 13 329–337. 10.1007/s10209-013-0313-4

[B115] Zhu L. Li H. Wang F. K. He W. Tian Z. (2020). How online reviews affect purchase intention: a new model based on the stimulus-organism-response (S-O-R) framework. *Aslib J. Inf. Manag.* 72 463–488. 10.1108/AJIM-11-2019-0308

