# Supplementary material for: The addiction behavior of short-form video app TikTok: The information quality and system quality perspective
Source: Front Psychol. 2022 Sep 6;13:932805. doi: 10.3389/fpsyg.2022.932805 (PMC9486470; doi:10.3389/fpsyg.2022.932805)
Supplement: Supplementary file 1 [file Data_Sheet_1.pdf]

Appendix

Table 1 Measurement

Construct	Dimension	Measurement Items	Sources
Information Quality	Conciseness	Short videos on ___ are usually short.<br>I spend very short time reading one message on ___.<br>I can understand the overall meaning of a __ in a short time.	(Lee & Kim, 2017)<br><br>(Zhang et al., 2016)
	Subscription	I can subscribe some interesting ___ users.<br>I can subscribe ___ whose video content are useful for my work or study.<br>I can gain useful information from ___ users that I subscribe.<br>Subscription can help me filter and distinguish information that I consider as misleading on ___.	
	Usefulness	The information on ___ is useful for work or study.<br>The information on ___ is valuable.<br>The information on ___ can satisfy my needs.<br>The information on ___ were informative.<br>___ provides me with interesting videos to my needs.	
System Quality	Flexibility	___ can be adapted to meet a variety of needs.<br>___ can flexibly adjust to new demands or conditions.<br>___ is versatile in addressing needs as they arise.	(Nelson et al., 2005)
	Integration	___ effectively integrates videos from different worldwide areas.<br>___ pulls together information that used to come from different places around the world.	

		The ____ effectively integrates videos from different areas of the international users.	
	Ease of Use	The _____ allows information to be readily accessible to me.<br>The ___ makes information very accessible.<br>___ is easy to use.<br>Learning how to use ___ TikTok is easy for me.<br>When I use ___ first time, I didn't have any problems.	
	Response Time	It takes short time for____ to respond to my request.<br>The _____ provides information in a timely fashion.<br>The _____ returns to answers my requests quickly.	
Enjoyment		I think that using ___ is enjoyable.<br>I think that watching ___ video is interesting.<br>I think that using ___ is pleasurable.<br>The actual process of watching videos on ___is pleasant.<br>___ provide me endless surprising experiences.<br>I feel curious when I watch ___ videos.	(Cao et al., 2020)
Concentration		When watching ______ video, I am deeply engrossed.<br>When watching _____ video, I am absorbed intensely.<br>When watching _____ video, I concentrate fully on it.<br>When watching _____ video, my attention is focused on the activity.	(Chen et al., 2017)
Time Distortion		Time goes by very quickly when watching ___.	

	I tend to lose track of time when watching ___. I tend to experience an insentient sense to time when watching ___.	(Novak et al., 2000)
TikTok addictive behavior	The idea of using ____ comes as the first thought on mind when wake up each morning. My life would be joyless hadn't there been ___. I feel distressed once I cease using ___ for a certain period. I feel missing something after stopping ___ for a certain period. I feel very vigorous upon watching ___ video regardless of the fatigues experienced. I can't help turning on ___ even when I'm not planning on using it. I find that I have been hooking on ___ longer and longer. I watch ____ videos for a longer period and spend more time than I had intended. I need to spend an increasing time on ___ to achieve same satisfaction as before. I try to spend less time on ___, but the efforts were in vain. I make it a habit to watch ___ videos and the sleep quality and total sleep time decreased. My recreational activities are reduced due to watching ___ videos. Watching ____ videos has exercised certain negative effects on my schoolwork or job performance. I find myself indulged on the ___ at the cost of hanging out with family members and friends.	(Yu & Fu-min, 2005)

	I feel aches and soreness in the back or eye discomforts due to excessive ___ use.
	I have slept less than 4 hours due to watching ___ more than once.
	I watch ___ videos for a longer period of time than I had intended.
	I feel tired on daytime due to late-night use of ___.
	I was told more than once that I spent too much time on ___.
	I feel missing something after stop using for a certain period of time.
